# Cook County Hospital

**Published:** 1869-08-15

**Authors:** C. Fenn

**Affiliations:** Chicago


### *Cook County Hospital.*

BY C. FENN, M. D., CHICAGO.

There is no "season" at the County Hospital. The student or practitioner may witness at any time a large number of instructive cases in each of the departments. The attractions are continual, and varied each day by the additions upon the register. The visitor who returns after a few weeks' absense will find almost an entire change has taken place in the occupants of the wards. The crowded condition of the hospital often necessitates the discharge of convalescents before they are fairly able to go, in order to make room for other emergency cases. Some afterward return to the dispensary for a continuance of treatment.

The clinics are held only from the first of October to the first of July; but the same gentlemanly attendants are on duty, and the same diversity and interest attaches to their charge during the remaining months as before. Pathology, of course, presents the highest attraction here, because of facilities of comparison; but, aside from this, pre-eminence cannot be given to any department. All are amply furnished with material for illustration.

The service is divided among the Medical Board, consisting of four attending surgeons, four attending physicians, two consulting surgeons, two consulting physicians, one gynecologist, one ophthalmologist, and one pathologist. The attending physicians and surgeons are each on duty three months of the year. The other members of the Board have no intermission of this term of service.

The following is the organization of attending surgeons: Prof. E. Powell, Dr. R. G. Bogue, Dr. T. D. Fitch, Dr. Charles G. Smith.

Attending Physicians: Prof. J. P. Ross, Prof. Thomas Bevan, Prof. H. A. Johnson, Dr. H. M. Lyman.

Consulting Surgeons: Prof. J. W. Freer, Dr. J. R. Gore.

Consulting Physicians: Prof. W. H. Byford, Dr. R. C. Hamill.

Gynecologist: Dr. H. Webster Jones.

Ophthalmologist: Dr. J. S. Hildreth.

Pathologist: Dr. Henry M. Lyman.

The whole number of patients treated in the hospital, exclusive of the outside dispensary, during the six months ending June 30, 1869, was: Surgical, 173; medical, 312; gynecological, 106; ophthalmological, 41. Total, 632.

